# Ancient schwannoma of the parapharynx causing dysphagia: a rare entity

**DOI:** 10.1308/003588412X13373405385737

**Published:** 2012-10

**Authors:** SI Sayed, P Rane, A Deshmukh, D Chaukar, S Menon, S Arya, AK D’cruz

**Affiliations:** Tata Memorial Hospital, Mumbai,India

**Keywords:** Schwannoma, Parapharynx, Oropharynx, Dysphagia

## Abstract

A schwannoma is a benign, encapsulated tumour that is derived from neural sheath (Schwann) cells. Approximately 25–40% of schwannomas occur in the head and neck. The most common site is the parapharyngeal space of the neck; oropharyngeal occurrence is extremely rare. Among the various histological types of schwannomas reported to date, the ancient (degenerative) variant is the most rare. To our knowledge, this is the first report of an ancient schwannoma in the parapharynx with an extensive oropharyngeal component causing dysphagia. Dysphagia was the prominent symptom because of the location and volume of the lesion. The tumour was excised via a transcervical approach.

Schwannoma (neurilemmoma) is a rare, benign, encapsulated tumour arising from neural sheath cells commonly seen in the extremities.^1^ The head and neck region accounts for approximately 25–40% of the total cases reported in literature.^1–3^ Schwannomas have been classified histologically into five types: common, plexiform, cellular, epithelioid and ancient.^4^ Among these, the ancient schwannoma is the most rare.^4^ The term ‘ancient schwannoma’ was first coined by Ackerman and Taylor in their review of 48 neurogenic tumours of the thoracic region.^5^ The term ‘ancient’ is given to cases showing typical neurilemmoma features but that are distinctive in terms of a long history and cellular architecture showing hypocellularity and a hyalinised matrix.^5,6^ Schwannomas with these degenerative changes can be misdiagnosed as sarcomas or as other forms of soft tissue neoplasms.

To date, ancient schwannomas presenting as neck masses have been reported by various authors (Table 1). In the literature, only a single patient has been reported to develop an ancient schwannoma in the parapharyngeal region.^6^ In our report, we present a case of an ancient schwannoma of the parapharynx and an extensive oropharyngeal component with a review of the current literature.

**Table 1:** Cases reported in the literature of ancient schwannomas presenting as mass in the cervical region

Authors	Site of ancient schwannoma
Bondy *et al*, 1996^7^	Submandibular gland
Moore *et al*, 1997^8^	Posterolateral pharynx
Saydam *et al*, 2000^3^	Cervical vagus
Walther *et al*, 2001^6^	Parapharyngeal space
Zachariades *et al*, 2001^9^	Cervical region
Hidaka *et al*, 2001^10^	Neck
Present case	Parapharynx

## Case history

**Figure 1:**
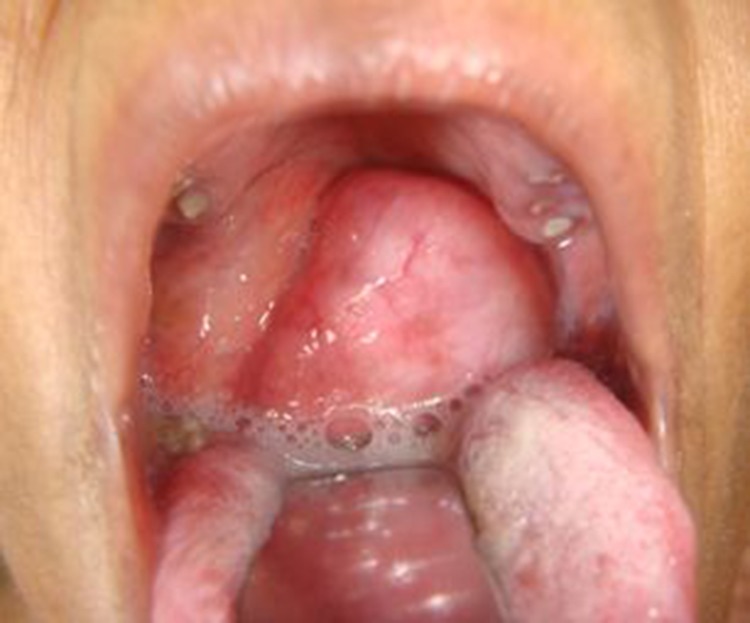
Intraoral examination showing a 3cm × 2cm mass obstructing the oropharynx completely with pooling of saliva

A 46-year-old man presented to a multidisciplinary tertiary cancer centre having suffered from a painless and progressively increasing mass in the right side of the submandibular region for the last 2 years. There had been an accompanying change in voice and dysphagia for the last five months. On clinical examination, he had a diffuse soft cystic swelling, measuring approximately 6cm × 5cm, anterior to the right sternocleidomastoid. The overlying skin was free. Intraoral examination revealed a large smooth bulge in the right lateral pharyngeal wall pushing the tonsil medially and causing narrowing of the oropharyngeal airway (Fig 1). The patient did not have any constitutional features of fever, weight loss or night sweats. The rest of the physical examination did not reveal any abnormality.

**Figure 2:**
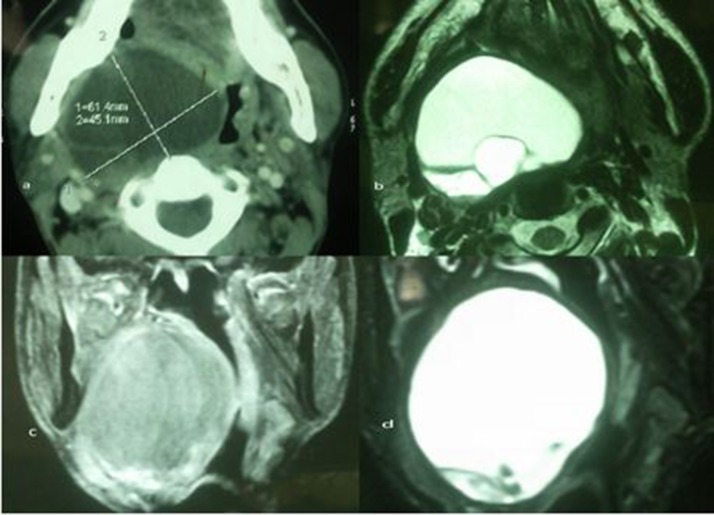
Computed tomography findings of the parapharyngeal ancient schwannoma: axial image showing a 6.1cm × 4.5cm mass in the right parapharyngeal space (top left), axial T2 weighted image showing the oropharyngeal mass with brightly hyperintense signal and few internal septa posteriorly (top right), coronal T2 weighted post-contrast image showing peripheral enhancement of solid components inferiorly (bottom left) and coronal T2 weighted image showing hyperintensity in the mass with septa and nodules seen inferiorly (bottom right)

The routine blood investigations were normal. Ultrasonography revealed a large oval anechoic lesion with multiple septa. On performing colour Doppler ultrasonography, there was no evidence of increased vascularity. Fine needle aspiration (FNA) was suggestive of a high grade pleomorphic malignant tumour but a formal biopsy was advised. T2 weighted magnetic resonance imaging (MRI) showed a 6.8cm × 6.3cm × 4.9cm hyperintense homogeneous cystic lesion in the right parapharyngeal space from the C1 to C4 levels that displaced the major vessels laterally (Fig 2). Planes around the swelling were well maintained. On gadolinium diethylene triamine pentaacetic acid contrast evaluation, the mass showed predominant peripheral enhancement with enhancement of intervening septa and solid components.

The differential diagnoses included second branchial cleft cyst, retropharyngeal abscess and schwannoma. The patient underwent surgical removal of the mass through a cervical approach under general anaesthesia. The procedure was without incident, with the mass being dissected easily from the surrounding tissues. Post-operative recovery was uneventful with no evidence of neural damage.

### Histological findings

**Figure 3:**
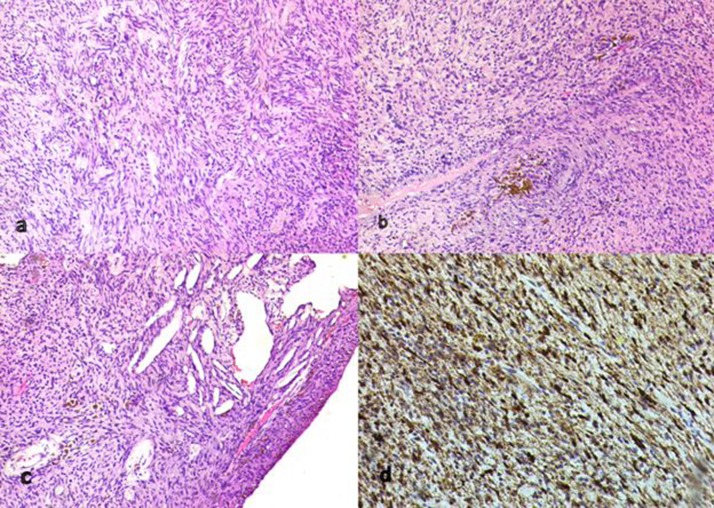
Microscopic images of the ancient schwannoma: spindle cells arranged in short fascicles with focal vague Verocay bodies (haematoxylin and eosin [H&E] stain, 100x magnification) (top left), schwannoma with focal collection of haemosiderin laden macrophages (H&E stain, 100x magnification) (top right), cholesterol clefts in ancient schwannoma (H&E stain, 100x magnification) (bottom left) and spindle cells immunoreactive for S100 protein (nuclear positivity) (indirect immunoperoxidase staining, 100x magnification) (bottom right)

The specimen consisted of a 7.5cm × 3.5cm × 2.5cm well circumscribed lesion with multiple cystic yellowish areas. The cyst wall thickness varied from 0.2cm to 0.5cm. On microscopy, the tumour was composed of short fascicles of spindle cells with eosinophilic cytoplasm, giving a syncytial appearance. Focally, the spindle cells were arranged in a vague palisade pattern (Verocay bodies). Cholesterol clefts, inflammatory cells and haemosiderin laden macrophages were seen sprinkled among the tumour cells. On immunohistochemistry, the spindle cells were immunoreactive to S100 protein. Overall, the features were those of an ancient schwannoma (Fig 3).

## Discussion

A schwannoma is a tumour originating from the Schwann cell of the nerve sheath. These tumours are typically slow growing and cause minimal symptoms to be detected late in the disease progression. They are characteristically solitary, well encapsulated and originate along the course of peripheral, cranial or sympathetic nerves.^11^ Due to their longstanding course, these tumours develop degenerative (ancient) changes in the form of cystic or myxoid degeneration.^12^ Although the head and neck region accounts for 25–40% of the schwannomas, it is extremely rare to develop an ancient schwannoma in the parapharyngeal region, with only one case being reported in the literature.^1–3,6^

These tumours present as a cystic mass in the neck region and therefore the differential diagnoses to be excluded are lymphadenitis (reactive/tuberculous/metastatic), lipoma, cystic hygroma, second branchial cleft cyst, carotid body tumour and laryngocoele. Inflammatory lymphadenitis will have a short disease history with tender neck swellings responsive to antibiotics. Tuberculous lymphadenitis will have classical matted swelling on palpation with the constitutional symptoms of fever and night sweats. Lipomas are superficial cystic swellings. Cystic hygromas are non-compressible cystic swellings with positive transillumination. Second branchial cleft cysts present usually as painless fluctuant masses in the lateral portion of the neck adjacent to the anteromedial border of the sternomastoid muscle, at the angle of the mandible. Carotid body tumours are firm, pulsatile neck masses.

These tumours exhibit characteristic histological features of an encapsulated lesion showing degenerative ‘ancient’ changes including large cystic/myxoid areas with occasional bizarre spindle cells and even a few mitoses.^12^ This histology usually displays a typical biphasic pattern with areas of hypercellularity (Antoni type A) and hypocellularity wherein a highly myxoid matrix predominates (Antoni type B).^13^ In the present case, the tumour was well encapsulated, and constituted both solid and cystic areas as described in the previous reports. It has been proposed that these tumours originate as a result of diffuse cellular overgrowth with increased vascularity, which is followed by decreased vascularity and resultant hyalinisation.^5^ These areas of hypercellularity seen on FNA are likely to be misdiagnosed as malignant.^3^

In our case, the FNA was suggestive of a high grade pleomorphic malignant tumour. These features of nuclear atypia and hyperchromatism seen on FNA in ancient schwannomas suggest a regressive phenomenon and should not be considered a sign of malignancy.^14^ Although FNA of ancient schwannomas may exhibit features of nuclear pleomorphism, nuclear inclusions, perivascular sclerosis, xanthomatous changes or nuclear atypia, the role of FNA is still questionable.^13,14^ In the current case, the diagnosis was only evident post-operatively.

To date, there have not been any major studies that have evaluated the role of radiological imaging in the diagnosis of ancient schwannomas. The single study done by Isobe *at al* that evaluated 24 schwannomas of the extremities showed that MRI is the most useful technique in evaluating ancient schwannomas.^15^ In the present case, the patient presented with a cystic neck mass and therefore ultrasonography of the neck was advised. This revealed a well defined hypoechoic mass with intervening septa. These findings can also be seen in conditions with non-inflammatory lesions such as malignant lymphadenopathy, lipomas, nerve sheath tumours, carotid body tumours, external laryngocoeles and cystic hygromas as well as with inflammatory lesions such as tuberculous nodes and abscesses. The various ultrasonography characteristics are described briefly in Table 2.

**Table 2:** Ultrasonography characteristics of neck masses

Pathology	Ultrasonography findings
Lymphomatous nodes	Round, hypoechoic with posterior enhancement
Metastatic nodes	Loss of hilar anatomy, round and hypoechoic
Reactive nodes	Hypoechoic, solid with presence of an echogenic hilum
Tuberculous nodes	Intranodal necrosis, matting and adjacent soft tissue oedema
Lipoma	Elliptical, parallel to skin surface, hyperechoic with linear echogenic lines
Cystic hygroma	Variable sized multiple cysts with thin walls and intervening septa
Nerve sheath tumour	Hypoechoic heterogeneous echo pattern
Carotid body tumour	Well defined solid non-calcified hypoechoic mass
Second branchial cleft cyst	Anechoic mass or predominantly hypoechoic, cystic mass with faint internal debris and posterior enhancement
Laryngocoele	Cystic mass, outside laryngeal framework with connection through thyroid membrane
Schwannoma	Hypoechoic mass, along a nerve and eccentric to its axis

As the ultrasonography did not reveal any useful diagnostic information, computed tomography (CT)/MRI was performed. Since the lesion was of a soft tissue, contrast enhanced MRI was chosen in view of its multiplanar capability and its better soft tissue contrast. In the literature, the CT appearance of a schwannoma has been described as a well circumscribed, inhomogeneous mass of low density, which can be explained by the following microscopic pattern: hypocellular areas (Antoni type B) adjacent to more cellular regions (Antoni type A) and cystic degeneration.^15,16^

Nowadays, MRI is considered to be the gold standard in the evaluation of ancient schwannomas. Schwannomas on T1 weighted MRI typically show a peripheral low signal intensity area and a high signal intensity area on T2 weighted images corresponding to the Antoni B areas.^15^ On gadolinium contrast injection, schwannomas show strong enhancement that is characteristic of the Antoni A area. On the other hand, a second branchial cleft cyst on T1 weighted images reveals a cyst that is isointense while recurrent infections may lead to hyperintense contents from increased protein concentration. T2 weighted images show hyperintense cysts without discernible walls. In order to differentiate this pathology from malignancy, one should take into account the clinicoradiological characteristic features, ie longstanding clinical history, circumference of a degenerative area and fibrous tumour capsules enhanced on MRI.

Surgical excision remains the mainstay of treating this benign condition although there is a lack of evidence to support this in view of the rarity of this disease entity.

## Conclusions

We have described a case of an ancient schwannoma originating in the parapharyngeal region. This type of tumour is uncommon in the head and neck region as well as in the parapharyngeal region. The diagnosis should be based on clinical findings of a longstanding painless cystic growth. MRI is the imaging modality to be relied on to clinch the diagnosis. FNA may be misleading histologically. Complete resection leads to complete cure of this benign disease.
